# How soil granulometry, temperature, and water predict genetic differentiation in Namibian spiders (*Ariadna*: Segestriidae) and explain their behavior

**DOI:** 10.1002/ece3.4929

**Published:** 2019-03-28

**Authors:** Erminia Conti, Christian Mulder, Anna Maria Pappalardo, Venera Ferrito, Giovanni Costa

**Affiliations:** ^1^ Department of Biological, Geological and Environmental Sciences University of Catania Catania Italy

**Keywords:** *Ariadna*, ecological factors, Kimura distance, Namib Desert, population divergence

## Abstract

The Namib Desert is a biodiversity hotspot for many invertebrates, including spiders. Tube‐dwelling spiders belonging to the *Ariadna* genus are widespread in gravel plains. These sit‐and‐wait predators share a particular behavior, as they spend their life in tunnels in the soil, surrounding the entrance of their burrow with stone rings. We investigated five spider populations taking into account environmental parameters, functional traits, and molecular data. We have chosen the temperature at the soil surface and at the bottom of the burrow, the air humidity, and the soil granulometry to define the environment. The chosen functional traits were the diameter and depth of the burrows, the ratio between weight and length, the thermal properties of their silks, and the number of ring elements. The molecular branch lengths and the evolutionary distance emerging from cytochrome oxidase I gene sequences summarized the molecular analysis. Our study highlights a strong coherence between the resulting evolutionary lineages and the respective geographical distribution. Multivariate analyses of both environmental and molecular data provide the same phylogenetic interpretation. Low intrapopulation sequence divergence and the high values between population sequence divergence (between 4.9% and 26.1%) might even suggest novel taxa which deserve further investigation. We conclude that both the Kimura distance and the branch lengths are strengthening the environmental clustering of these peculiar sites in Namibia.

## 1 INTRODUCTION

Namibia is one of the world's drylands at greatest risk of large rainfall changes, and urgent actions are needed to prepare the country for further decreases in rainfall (Chadwick, Good, Martin, & Rowell, 2016). Quaternary reconstructions of aridity and trade‐wind strength in southwestern Africa clearly show that eolian erosion in Namibia is remarkably high and the long‐term combination between such an extreme erosion, huge aridity and direct and intense solar radiation is a major environmental driver for any living organism. These factors also produced a very high level of endemism (20% of described species are endemic to Namibia) and stimulated extraordinary adaptive responses to the environment that hosts them. In particular, the Central Namib Desert is a biodiversity hotspot for many vertebrates and invertebrates (Prendini & Esposito, 2010; Simmons, Griffin, Griffin, Marais, & Kolberg, 1998). Moreover, it is estimated that 11% of arachnids in Namibia are endemic and about 90% of the occurring invertebrates might not have been described yet (Ministry of Environment & Tourism, 2014).

Spiders have been also increasingly used as nonconventional terrestrial bioindicators (Conti et al., 2018; Wilczek, 2017). Their ability to survive under extreme conditions has allowed them to colonize several ecosystems. For instance, they can easily mitigate the intense heat by living below the surface (Lawrence, 1962). There are no biogeographical zones where spiders are not present and consequently, their ecological role cannot be underestimated (Nyffeler & Birkhofer, 2017). About 25 years ago, Costa, Petralia, Conti, Hänel, and Seely (1993) recorded the existence of numerous and large tube‐dwelling spider populations on the gravel plains of the Namib Desert. These populations were identified as belonging to the genus *Ariadna* (Segestriidae).

The genus *Ariadna* (Audouin, 1826), belonging to the Synspermiata spider family Segestriidae (Michalik & Ramírez, 2014), has an almost worldwide distribution. The taxonomy of Segestriidae seems to be quite chaotic (World Spider Catalog, 2018). This makes an integrated approach based on molecular data as well as ecological features and functional traits the best response to highlight how different populations can face different environmental conditions especially in such extreme habitats. Elsewhere, studies on functional traits like body size have been carried out in order to link phylogeny and ecosystem services as well as to identify phylogenetic relationships between species (Cavender‐Bares, Kozak, Fine, & Kembel, 2009; Mulder et al., 2013). As far as we know, functional traits like the depth and diameter of burrows have never been taken into account for taxonomic surveys yet.

A global revision of the *Ariadna* genus becomes a hard task and to date, only some revisions limited to America have been carried out (Beatty, 1970; Giroti & Brescovit, 2018; Grismado, 2008). In particular, taxonomy and distribution of the *Ariadna *genus in Namibia (Lawrence, 1928; Purcell, 1904, 1908; Strand, 1906) are rather confusing, sometimes even without the sampling location of the specimens. Species that are recorded for Namibia are *Ariadna insularis* (Purcell, 1904,^1908^), *A. viridis* (Strand, 1906), *A. masculina* (Lawrence, 1928), but taxonomical identification at species level was hard in the Central Namib Desert (Eryn Griffin *personal communication*). The DNA barcoding method based on cytochrome oxidase I gene appears to be particularly useful for a fine‐tuned discrimination when morphological analysis is lacking (Čandek & Kuntner, 2015; Hebert & Gregory, 2005). In particular, DNA barcode reference libraries have been built for spiders both at regional scale (e.g., Astrin et al., 2016; Blagoev et al., 2016; Gaikwad, Warudkar, & Shouche, 2017; Naseem & Tahir, 2018) and at broader geographical scale (e.g., Robinson, Blagoev, Hebert, & Adamowicz, 2009; Barret & Hebert, 2005; Coddington et al., 2016). The purpose of this work is to understand to what extent ecological factors in a hyperarid environment might have led to a specific differentiation within the genus *Ariadna* with site‐specific behavioral features.

## 2 MATERIALS AND METHODS

### 2.1 Fieldwork

**Figure 1:**
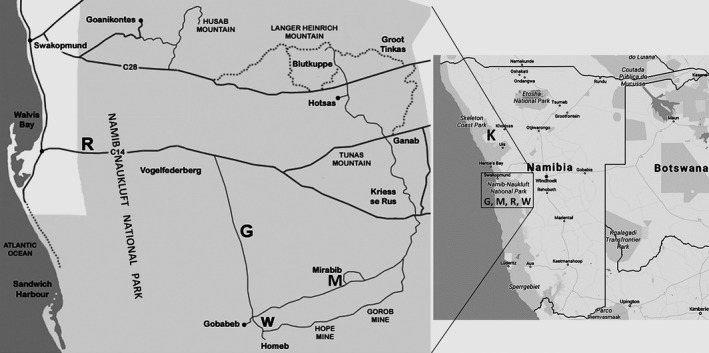
Location of the sampled areas. The enlargement shows the research stations inside the Namib Naukluft Park

Adult specimens of *Ariadna* spiders were collected from March 25 to April 21, 2012. Namibia can be easily classified into three terrestrial biomes Desert, Karoo, and Savanna (Mendelsohn, Jarvis, Roberts, & Robertson, 2002). Of our research stations, four (G, M, R, W) are within the Desert biome and one (K) is in the Savanna biome (Figure 1). The sites investigated can be described as follows:

The G site (23°19.0′38.4″S, 15°2.0′23.3″E) lies in the Central Namib Desert, 56 km from the Atlantic coast and 25 km from the Gobabeb Research and Training Station. Fog is less frequent, and humidity is lower than in the areas closer to the coast but wind can blow strongly (Kaseke, Wang, & Seely, 2017; Viles, 2005; Wentworth, 1922).

The K site (20°25.0′53.1″S, 14°20.0′44.9″E) is a dry savannah area of the Great Escarpment, external to the Namib desert, about 115 km from the Atlantic coast and 71 km from Khorixas.

The M site (23°32.0′53.2″S, 15°8.0′23.8″E), in the Central Namib Desert, is 70 km from the Atlantic coast and 17 km from Mirabib. Like G, local precipitation is pulsed and unpredictable (Agnew, 1997; Jürgens, Burke, Seely, & Jacobsen, 1997) and fog is sparse if compared to the coastal areas. The mean annual humidity is lower, whilst the mean annual temperature is higher than else (Seely, 1987).

The R site (23°0.0′32.7″S, 14°43.0′38.0″E) is a part of the famous lichen area in the Namib Desert, 22 km from the Atlantic coast and 10 km from the Rooikop airport. A thick fog daily and strong winds are typical of this site (Costa & Conti, 2013; Seely, 1987; Viles, 2005) where a gravel plain consisting of a gravelly sandy sediment with small quartz pebbles, rich in lichens.

The W site (23°36.0′32.9″S, 15°10.0′2.7″E), characterized by the presence of some specimens of the dwarf gymnosperm *Welwitschia mirabilis*, is located 72 km from the Atlantic coast and 14 km from the Gobabeb Research and Training Station. The W site includes a 3–20 m wide river dry bed that is a dry tributary of the Kuiseb River (Henschel & Seely, 2000).

We measured the temperature at the soil surface and collected the spiders as described in the next subsection.

### 2.2 Spider sampling

**Figure 2:**
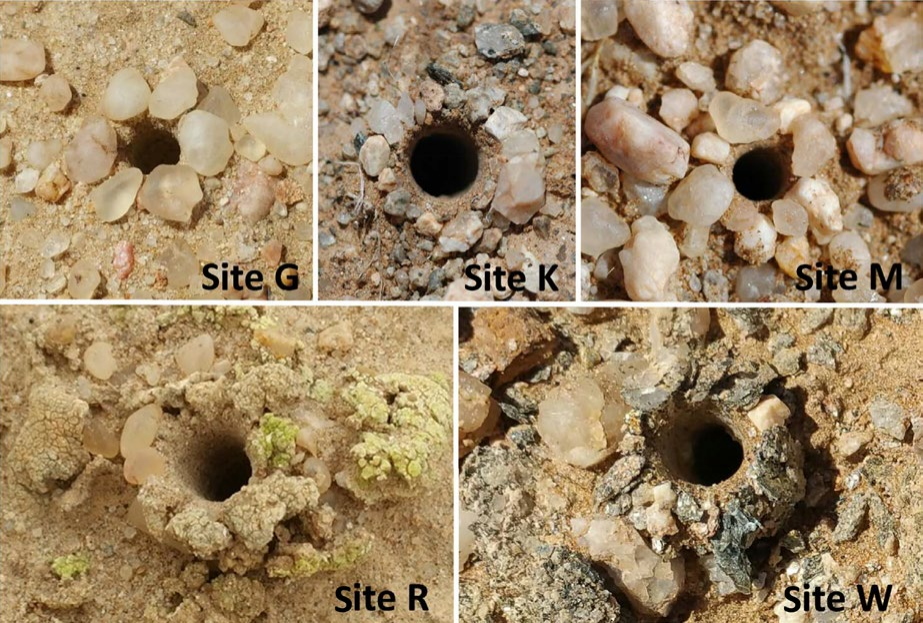
*Ariadna* burrow features from the investigated sites (clockwards: the G, K, M, W, and R sites). The burrow rings of the G and M sites include most commonly 6–7 quartz stones, similar in size, shape, and color and arranged in only one layer, but M rings are less regular than G ones. In the K site, the burrow rings include numerous small stones, placed in a single layer and differing in size, shape, and structure. In the R site, the burrow rings include 6–15 quartz stones mixed with pieces of lichens and arranged in one to four strata to shape a typical turret. Finally, in the W site, the burrows are dug on the slopes of the tributary and the ring stones are numerous, irregular and arranged in up to four strata

A total of 88 adult *Ariadna* specimens (about 20 specimens per site) were collected from their own burrows during our 2012 survey. For each specimen, we estimated weight using a Sartorius balance (model CPA225D) and total body length using a Borletti caliper (measurement error of 0.02 mm). We also measured in field the depth below surface and diameter of entrance burrows of each spider. After measurements, spiders were individually placed alive in Falcon tubes (50 ml) filled by half with sand collected from the burrow. They were kept at temperature of 20–22°C, approximately 67%–69% relative humidity, and photoperiod corresponding to that of the sampling area (i.e., 13 hr 15’L:10 hr 45’D) until their shipping to Italy for molecular analyses. Figure 2 shows the morphological difference between the burrows of *Ariadna* spiders. The individual burrow of these spiders is conspicuously different, due to its vertically oriented tube, internally covered with silk, and with a circular entrance surrounded by a stone ring, with sometimes lichen bits (Costa, Petralia, Conti, & Hänel, 1995), and the features of the burrow rings vary according to population and habitat (Costa et al., 2000).

### 2.3 DNA barcode sequencing

Total genomic DNA was extracted from 38 entire spider specimens using the DNeasy Blood & Tissue Kit (Qiagen, Milan, Italy) according to the manufacturer's instructions. COI sequences were obtained using the primers HCO2189 (5‐TAA ACT TCA GGG TGA CCA AAAAAT CA‐3) and LCO1490 (5‐GGT CAA CAA ATC ATA AAG ATA TTGG‐3) (Folmer, Black, Hoeh, Lutz, & Vrijenhoek, 1994). All PCR amplifications were carried out in 25 µl total volume using approximately 50 ng of the isolated DNA as a template. In addition, each PCR contained 1X Taq DNA polymerase buffer (supplied by the respective Taq DNA polymerase manufacturer), 1.5–2 mM of MgCl_2_, 200 mM of each dNTP, 10 pmol of each primer and 0.5 U of Taq DNA polymerase (Platinum Taq DNA polymerase, Invitrogen). An initial denaturation at 94°C for 15 min was followed by 35 cycles (denaturation at 94°C for 30 s, annealing at 51°C for 1 min and extension at 72°C for 1 min) and a final extension at 72°C for 10 min. Negative controls were included in all PCR runs to ascertain that no cross‐contamination occurred. Double‐stranded products were checked with agarose gel electrophoresis, purified with the QIAquick PCR purification kit (Qiagen) and subsequently sequenced in the forward and reverse direction by Genechron (http://www.genechron.it/index.php/sangersequencing) using an ABI Prism 3100 automated sequencer (Applied Biosystems). Sequences were carefully checked and deposited in GenBank (http://www.ncbi.nlm.nih.gov/genbank). In our case, we built the COI barcode sequence reference library only from female specimens of five populations (Table 1).

**Table 1:** Specimens from spider populations used for the COI molecular analysis. The complete sequences described in Materials and Methods have been deposited in GenBank under Accession Numbers MK294181 to MK294206

Region	Site ID	Sample ID	H	GenBank accession no
Gobabeb	G	G1	Hap 1	MK294181
	G	G2	Hap 2	MK294182
	G	G3	Hap 5	MK294185
	G	G4	Hap 1	MK294181
	G	G5	Hap 3	MK294183
	G	G6	Hap 6	MK294186
	G	G7	Hap 4	MK294184
	G	G8	Hap 2	MK294182
Rooikop	R	R1	Hap 8	MK294188
	R	R2	Hap 9	MK294189
	R	R3	Hap 7	MK294187
	R	R4	Hap 7	MK294187
	R	R5	Hap 10	MK294190
	R	R6	Hap 9	MK294189
	R	R7	Hap 9	MK294189
	R	R8	Hap 7	MK294187
Mirabib	M	M1	Hap 11	MK294191
	M	M2	Hap 14	MK294194
	M	M3	Hap 15	MK294195
	M	M4	Hap 12	MK294192
	M	M5	Hap 11	MK294191
	M	M6	Hap 11	MK294191
	M	M7	Hap 13	MK294193
Welwitschia	W	W1	Hap 16	MK294196
	W	W2	Hap 16	MK294196
	W	W3	Hap 19	MK294199
	W	W4	Hap 17	MK294197
	W	W5	Hap 18	MK294198
	W	W6	Hap 19	MK294199
	W	W8	Hap 16	MK294196
Khorixas	K	K1	Hap 20	MK294200
	K	K2	Hap 21	MK294201
	K	K3	Hap 23	MK294203
	K	K4	Hap 26	MK294206
	K	K5	Hap 24	MK294204
	K	K6	Hap 22	MK294202
	K	K7	Hap 23	MK294203
	K	K8	Hap 25	MK294205

### 2.4 Genetic analysis

The chromatograms obtained were edited using BioEdit (http://www.mbio.ncsu.edu/bioedit/bioedit.html) to generate a consensus sequence for each specimen. The DNA sequences were aligned using the ClustalX (Thompson, Gibson, Plewniak, Jeanmougin, & Higgin, 1997) tool incorporated into MEGA v5.0 software (Tamura, Stecher, Peterson, Filipski, & Kumar, 2013). Sequences were trimmed to 617 bases and were compared with other spider mitochondrial genomes using BLAST (http://www.blast.ncbi.nlm.nih.gov/Blast.cgi) to confirm the identity of the obtained fragments (the 5’ COI region). The sequence divergences within and between Operational Taxonomic Units (OTUs) were calculated using the distance model Kimura‐2‐Parameters (Kimdist) and the bootstrapping proportion (1,000 iterations) was computed according to Hillis and Bull (1993). The Kimdist dendrogram using the Neighbor‐Joining algorithm, as clustering method for analysis of barcoding data (Hajibabaei, Singer, Hebert, & Hickey, 2007), was generated using as outgroups all the Segestriidae deposited in GenBank when accessed on December 13, 2018. Three outgroups were deposited and are* Ariadna insidiatrix *Audouin (GenBank Acc. Number: KY017904) and the closely related *Citharoceps fidicina* Chamberlin (GenBank Acc. Number: FJ607555), and finally *Segestria bavarica* C. L. Koch (GenBank Acc. Number: KY268449).

### 2.5 Statistical analysis

For statistical analyses, we considered six environmental variables: temperature at soil surface (Tsurf), temperature at the bottom of the burrow (Tdepth), humidity, and granulometry (i.e., percentage of gravel, silt, and sand). In addition, we examined five functional traits: diameter of the burrow entrance (DIA), burrow depth (DEPTH), body‐mass index (BMI, calculated as ratio between weight and length of each spider), thermal properties of the silk considered as total normalized enthalpy of melting (DSC‐Hm as in Conti et al., 2015), and total number of ring elements at the higher stratum (RINGS). Finally, we used two molecular variables: the value of branch lengths (Tree‐BL, i.e., the sum of units of substitutions per site of the sequence alignment) and the evolutionary distance emerging from the Kimdist distinguishing between *transitions* from purine to purine or from pyrimidine to pyrimidine and *transversions* from purine to pyrimidine or from pyrimidine to purine (Kimura, 1980).

The normality of the data was tested by a Shapiro–Wilk test (Shapiro & Wilk, 1965) and the homogeneity of variance by a Levene's test (Levene, 1960). To test for differences between populations, we used a one‐way ANOVA (using Δ*T* calculated as difference between Tsurf and Tdepth as temperature variable). Post hoc comparisons were conducted using the Dunnett's test (Dunnett, 1964) for the variables where ANOVAs were significant. To test for differences between sites, we used a Pearson's correlation using burrows’ VOL as functional variable fully predicted by the trait “weight” of the burrow‐inhabiting spiders. To identify which variables weighed more in separating spider populations, we performed two different principal component analysis (PCA with Varimax): a first PCA with environmental variables alone and a second one with all the variables examined. The PCA is able to synthesize successfully abiotic data and to put them in an ordination based on environmental variables alone. The significance limit for the statistical analyses was *p* = 0.01. ANOVA was performed using the SPSS package for Windows (v. 21.0), whilst for PCA analysis and Pearson's correlation we used XLSTAT 2018.2 for Windows 10.

## 3 RESULTS AND DISCUSSION

### 3.1 Soil and temperature

**Figure 3:**
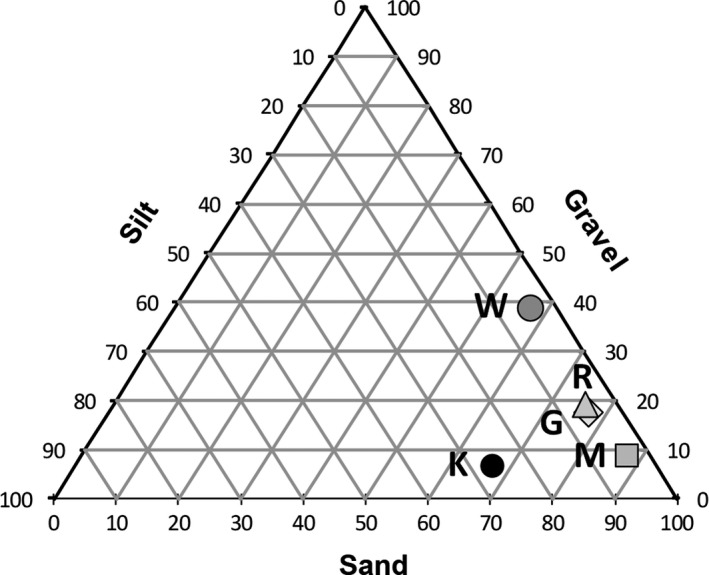
Ternary diagram showing the particle size distribution (%) in the investigated sites

As expected, the soil granulometry of the investigated biomes is different, with the sites G, M, R, and W close to each other and rather far away from K, where the silt component is about 5‐times higher than G (Figure 3). The sandy component of M is by far the highest among our sites (88.05%). The temperature of the soil surface at the G site during our sampling was between 36 and 48°C, at the M site between 36 and 49°C (hence close to G), at the R site between 20 and 39°C (the lowest of our five sites), and at the W site between 21 and 46°C. Also the K site, a dry savannah area of the Great Escarpment, is characterized by high temperatures between 23 and 45°C.

### 3.2 Analysis of variance

**Figure 4:**
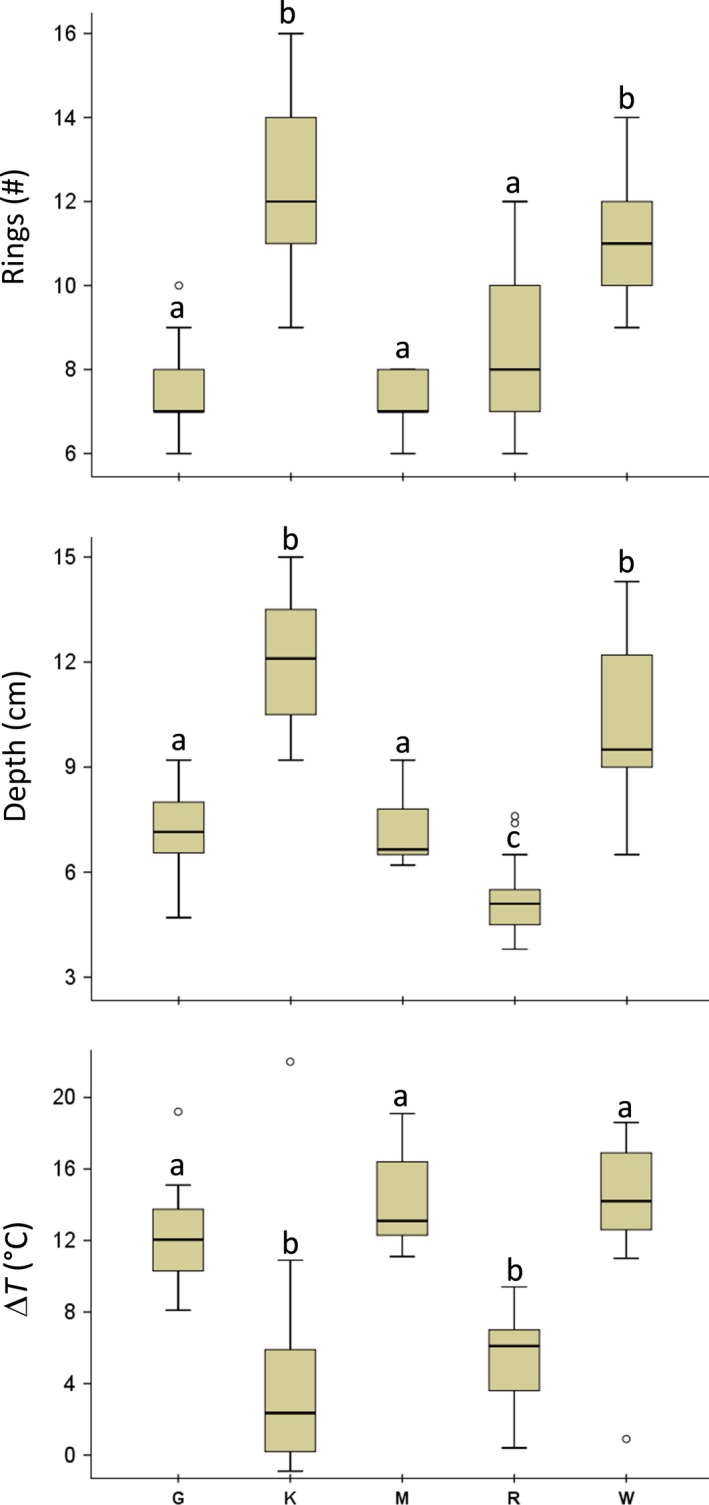
Boxplot of variables showing significant difference between populations according to the Dunnett ANOVA test (RINGS: total number of ring elements; DEPTH: burrow depth; Δ*T*: difference between Tsurf and Tdepth)

The statistical differences between populations according to ANOVA (Figure 4) have been significant for RINGS (*F* = 35.594; *p* < 0.001), DEPTH (*F* = 77.278; *p* < 0.001) and ΔT (*F* = 25.424; *p* < 0.001), as expected from the remarkably diverse environmental conditions. The four sites within the Namib Naukluft National Park (G, M, R and W) are strongly affected by the Benguela Current that causes high values of humidity especially during nighttime and early in the morning, with R as closest to the coast (Van Zinderen Bakker, 1975; Wefer, Berger, Siedler, & Webb, 1996). Moreover, the sites G and M are mainly composed of sand, and therefore, their soils are highly permeable. Local humidity and granulometry are probably why these spider burrows are relatively deeper compared to K and W ones. Low depths imply spiders make little silk, and therefore, they do not need many elements to stabilize their home (Figures 2 and 4).

R depicts rather mild climatic conditions (moderate temperature and high humidity as in Seely, 1987) which drive spiders to dig short burrows. A widespread growth of ground‐dwelling lichens is characteristic for this site, and the behaviour of the spiders seems to relate to this peculiar environmental condition. For instance, the spiders in R form turrets and use lichens in the construction of their burrows. This is probably due to their shallow burrows and a lesser need to amplify the vibrations of web‐captured preys since the spider is close to the surface and the lichenophagous fauna is abundant. Site W, in contrast to the other three sites inside the Namib Naukluft National Park, is characterized by a raw soil texture (plenty of gravel, 37.10%–40.89% in comparison to a sand fraction of 54.14%–59.35%).

The burrows from site K show the semi‐arid conditions of the savanna where high temperature compels spiders to construct their homes deep in the soil, guaranteeing optimal microclimatic conditions for adults, eggs, and spiderlings. The relevance of silt in the soil granulometry (plenty of silt, 24.04%–28.57% in comparison to a sand fraction of 64.64%–69.71%) can be seen according to us as an indirect geological evidence of an ancient erosion process that bring us to speculate that K spiders appear on first and afterward other populations evolved. This long‐history of continuous soil erosion changed the site‐specific environmental responses of all our spiders, as suggested by the largest number of items of the rings that are also different in size, shape, and structure (shown in Figure 2 and analyzed in Figure 4, upper panel).

### 3.3 Multivariate analysis

**Figure 5:**
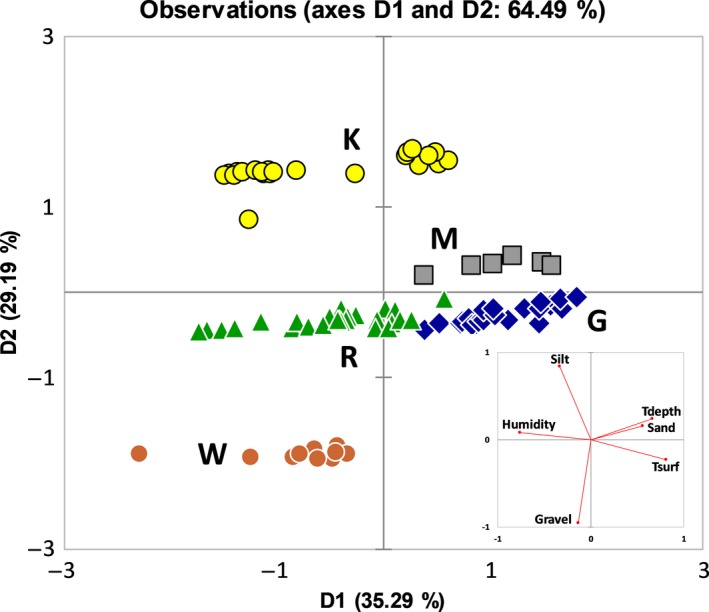
Principal component analysis plot (PCA after Varimax rotation) showing the scattered data based on environmental variables alone. On the right, plot of the variables. Plotting dimension 1 (D1) against dimension 2 (D2) revealed site clustering

**Figure 6:**
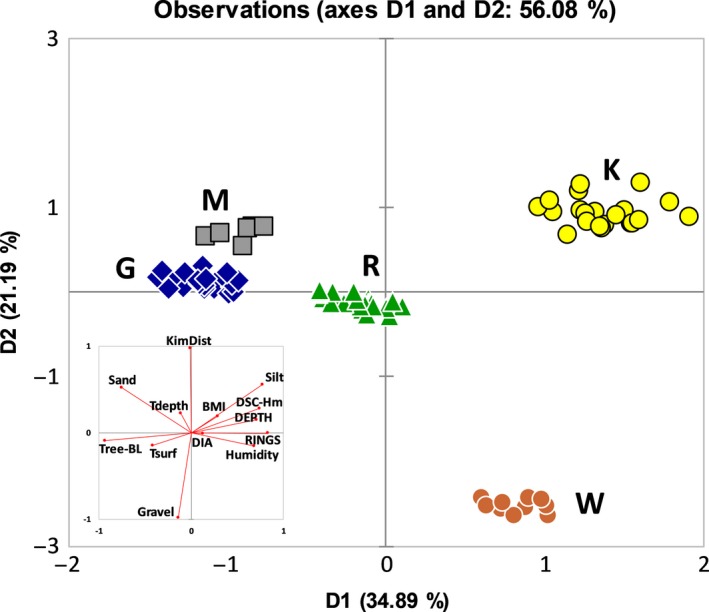
Principal component analysis plot of PC1 and PC2 (after Varimax rotation) showing the scattered data based on environmental, functional, and molecular variables. On the left, plot of these variables. Plotting dimension 1 (D1) against dimension 2 (D2) after entering the functional and the molecular data revealed much better site clustering than in the previous Figure 5 despite its slightly higher predictive power (64% vs. 56%)

As mentioned in the Materials and Methods, we performed a Varimax rotation of the principal components (PC) to maximize the independence between them (Figures 5 and 6). Squared cosines of the environmental variables after the Varimax rotation were the largest for Tdepth, Tsurf, humidity, and sand percentage for the first dimension (0.442, 0.656, 0.580, and 0.312, respectively) and for the percentages of silt and gravel for the second dimension (0.704 and 0.906, respectively).

After adding molecular data and functional traits to microclimate and soil granulometry, the squared cosines of all the variables were the largest for BMI, DIA, DEPTH, Tree‐BL, Tsurf, humidity, silt, sand, DSC‐Hm, and RINGS for the first dimension (0.084, 0.015, 0.498, 0.867, 0.171, 0.462, 0.596, 0.571, 0.551, and 0.688, respectively) and for KimDist, Tdepth, and gravel percentage for the second dimension (0.961, 0.052, and 0.959, respectively).

Therefore, multivariate analysis clearly shows how the sites can be easily distinguished based on environmental data alone (Figure 5). In particular, after the Varimax rotation, the first dimension (D1) is explained by microclimate (79.3% as we considered Tsurf and Tdepth separately avoiding Δ*T* to minimize redundancy; Table 2), whilst the second dimension (D2) is almost fully explained by granulometry (93.4%; Table 2).

**Table 2:** Component score coefficients and contribution of the environmental variables (%) for dimension 1 (D1) and dimension 2 (D2) after Varimax rotation (Tsurf: temperature at soil surface; Tdepth: temperature at the bottom of the burrow; humidity; silt, sand, and gravel)

	D1	D2	D1 (%)	D2 (%)
Tdepth	0.325	0.165	20.882	3.348
Tsurf	0.376	−0.098	30.990	2.910
Humidity	−0.359	0.017	27.405	0.378
Silt	−0.124	0.469	5.144	40.187
Sand	0.271	0.113	14.716	1.429
Gravel	−0.101	−0.552	0.863	51.747

When all variables have been taken into account, the explanatory contribution of microclimate becomes quite small (just 10.2% for humidity) on the first dimension (Table 3), whilst functional traits become relevant (40.5%). After the Varimax rotation of the PCs of all variables (Figure 6), the first dimension (D1) is, besides by genetics (Tree‐BL 19.1%; Table 3), mostly explained by rings, silk, and burrows’ depth (15.2%, 12.1% and 11%, respectively), whilst the second dimension (D2) is, besides by genetics again (KimDist 34.9%; Table 3), fully explained by granulometry (56.2%; Table 3).

**Table 3:** Component score coefficients and contribution of all the variables (%) for dimension 1 (D1) and dimension 2 (D2) after Varimax rotation (BMI: body‐mass index calculated as ratio between weight and length of each spider; DIA: diameter of the burrow entrance; DEPTH: burrow depth; Tree‐BL: the value of branch lengths expressed as the sum of units of substitutions per site of the sequence alignment; KimDist: the evolutionary distance emerging from the Kimura distance; Tsurf: temperature at soil surface; Tdepth: temperature at the bottom of the burrow; humidity; silt, sand, and gravel; DSC‐Hm: thermal properties of the silks considered as total normalized enthalpy of melting; RINGS: total number of ring elements)

	D1	D2	D1 (%)	D2 (%)
BMI	0.057	0.060	1.849	1.392
DIA	0.028	−0.008	0.333	0.001
DEPTH	0.153	0.024	10.980	0.820
Tree‐BL	−0.206	0.008	19.118	0.301
KimDist	−0.045	0.365	0.001	34.896
Tdepth	−0.036	0.090	0.287	1.904
Tsurf	−0.087	−0.035	3.777	0.745
Humidity	0.160	−0.088	10.198	0.863
Silt	0.150	0.173	13.149	11.321
Sand	−0.194	0.229	12.586	10.075
Gravel	0.013	−0.358	0.415	34.802
DSC‐Hm	0.155	0.072	12.143	2.881
RINGS	0.187	−0.037	15.163	0.000

Looking to the PCA of Figure 5 it is evident that on average the sites are much more scattered due to heterogeneity of the considered environmental variables. In particular, G, M, and R are stretched horizontally along the first dimension due to a strongly different microclimate. On the contrary, K and W stretched vertically along the second dimension due to highly different granulometry (Figure 3).

Surprisingly, using all variables together, the multivariate ordination of our *Ariadna* specimens is much less scattered than using environmental variables alone (compare Figure 6 with Figure 5). Comparing Figure 5 with Figure 6, G and M (and to a lesser extent R) remain close to each other, reflecting the aforementioned consideration that being G and M composed of sandy soils, their spider populations show similar behaviour in construction of their burrows (relatively deep burrows and low number of elements forming the rings). KimDist and Tree‐BL are clearly strengthening the clustering of the sites in Figure 6. Hence, molecular data contribute to the differentiation between our sites both in the first as in the second dimension.

### 3.4 DNA Barcoding analysis

**Figure 7:**
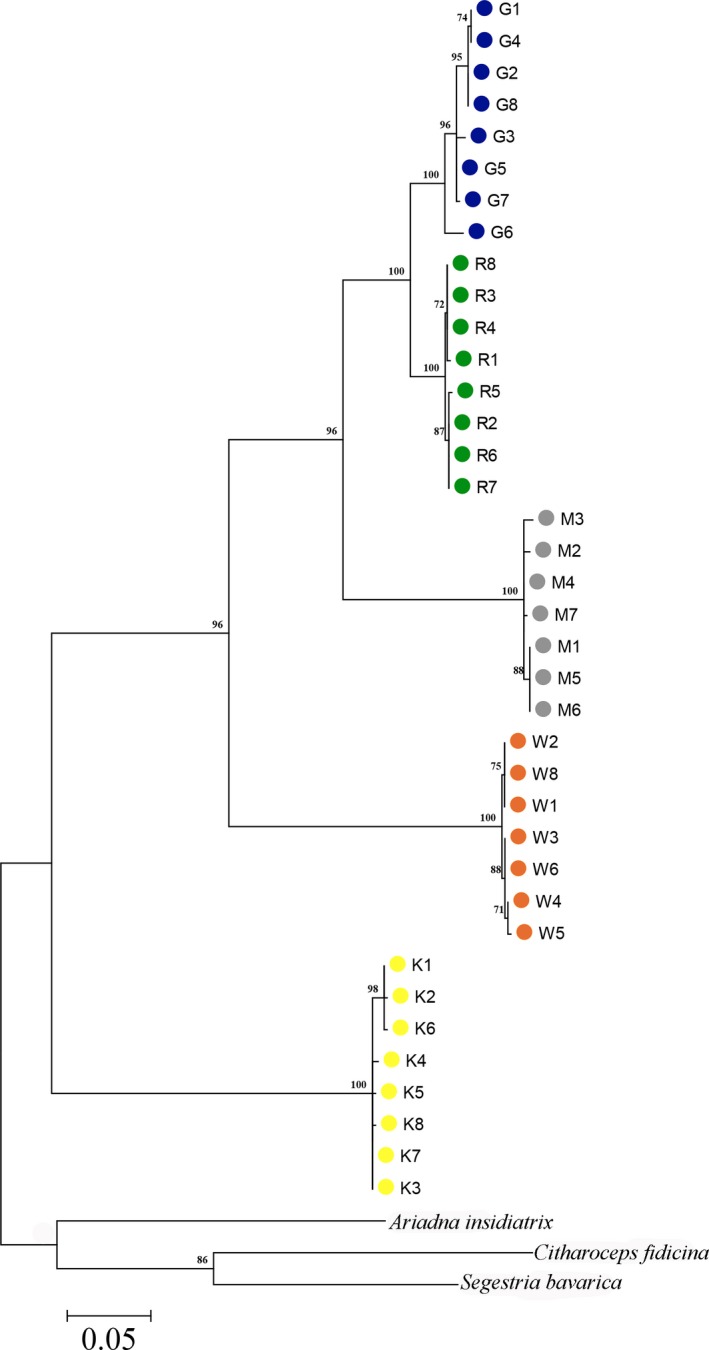
Neighbor‐joining (NJ) tree generated by COI sequences of our *Ariadna* spiders and all the Segestriidae deposited in GenBank when accessed December 13, 2018. The bootstrap support (>70%) for each clade is indicated above the branches. The bar indicates the distance scale

The fragment of 617 bp of COI sequences investigated corresponds to the barcode region proposed by Hebert, Cywinska, Ball, and deWaard (2003) and Hebert, Ratnasingham, and deWaard (2003) for species identification. The comparison of sequence through BLAST queries confirmed the identity of the obtained fragments for the spider samples. The analyses of 38 sequences led to the identification of 26 haplotypes forming five clusters all supported by high bootstrap values (>70%) in the NJ tree (Figure 7). Each cluster included the COI sequences of a single population. Only private haplotypes have been found in each population (Table 1). Furthermore, the low intrapopulation sequence divergence below 3%, and the high values between population sequence divergence among 4.9% and 26.1% (Table 4) fall within the intraspecific and interspecific range values, respectively, obtained for COI sequence of spiders (Barret & Hebert, 2005). Therefore, these high divergence values between all populations investigated (on average 18%) could reflect the presence of cryptic species in this group of spiders. The less than 3% divergence values between haplotypes within each population confirm the presence of “barcoding gap” as a delimiting criterion for species differentiation (Hebert, Penton, Burns, Janzen, & Hallwachs, 2004; Meyer & Paulay, 2005).

**Table 4:** Phylogenetic group averages between KimDist matrix

	W	M	G	R	K
W		0.021	0.019	0.020	0.022
M	0.195		0.016	0.016	0.024
G	0.185	0.137		0.009	0.020
R	0.183	0.131	0.049		0.020
K	0.236	0.261	0.215	0.210	

The phylogenetic tree shows a remarkable similarity between molecular data (Figure 7) and PCAs with either environmental variables (Figure 5) or all the variables (Figure 6), being G, M, and R spiders always close to each other. Given the positions in our phylogenetic tree of the Mediterranean outgroup (*A. insidiatrix*) and of the American outgroup (*C. fidicina*, cf. Chamberlin, 1924), we are not proposing any new species for the three sites groups of K, W, and G‐M‐R although we strongly confirm that these Namibian taxa must be all species belonging to the worldwide distributed *Ariadna* genus as described by Beatty (1970). Summarizing, this remarkable coherence between microclimate, behavioral traits and evolutionary lineages for our five *Ariadna* populations makes clear how easily behavioral ecology provides the right perspective to recognize different taxa of spiders and possibly other invertebrates.

## CONFLICT OF INTEREST

None declared.

## AUTHOR CONTRIBUTIONS

EC and GC designed the study; EC and GC sampled the material; EC measured the spider traits in situ; AMP and VF generated the COI data, which were analyzed by AMP; EC and CM performed the statistical analysis; EC, AMP, and CM led the data quality assessment and interpreted the results. EC and CM led manuscript writing.

## Data Availability

COI sequences were obtained using the primers HCO2189 (5‐TAA ACT TCA GGG TGA CCA AAAAAT CA‐3) and LCO1490 (5‐GGT CAA CAA ATC ATA AAG ATA TTGG‐3). Final DNA sequence assembly deposited in GenBank with the accession numbers as in Table 1.
